# Medical Miscellanies

**Published:** 1818-05

**Authors:** 


### PART IV.

### MEDICAL MISCELLANIES.

Quicquid agunt Homines.

### *Sketch of the Life and Character of the late* BENJAMIN RUSH, M. D. LL. D. *Professor of the Institutes and Practice of Medicine in the University of Pennsylvania, &c. &c.*

(From the American Medical and Philosophical Register.)

BENJAMIN RUSH was born near Bristol, in the state of Pennsylvania, on the 5th of January, 1745. His ancestors belonged to the society of Quakers, and were of the number of those who followed the celebrated William Penn to Pennsylvania, in the year 1683: his grandfather, James Rush, resided on his estate near Philadelphia, and died in the year 1727. His son, who was the father of the subject of these memoirs, inherited both his farm and his trade, which was that of a gun-smith. He died while Benjamin was yet young. His widow, a most excellent woman, upon whom the education of young Rush was thus necessarily devolved, placed him, at an early age, under the direction of the late Rev. Samuel Finley, at West Nottingham, in Chester county, Pennsylvania, by whom he was taught the rudiments of classical knowledge. Dr. Finley, afterwards better known as the president of Princeton College, New Jersey, was an able scholar and faithful teacher, and, being also related to Mrs. Rush, may be supposed to have paid great attention to the improvement of his young pupil. But whatever may have been the assiduity with which his education was directed by his preceptor, he possessed an ardent desire for knowledge, and was most unwearied in the pursuit of it.

From the academy of Dr. Finley he was removed to the college of Princeton, where he finished his classical edution, and was admitted to the degree of A. B. in 1760, when he had not yet completed his sixteenth year. He was now left to choose a profession, and in the choice which he made he doubtlessly was actuated by conscientious motives. He seems to have fully known his own character, and to have formed a proper estimate of his talents, and by applying them to the science and practice of medicine, to have been desirous of doing all possible good to the family of mankind. That he was directed by these motives, may be inferred from his own opinion of the utility of medicine. "So great," says he, "are the blessings which mankind derive from it, that if every other argument failed to prove the administration of a providence in human affairs, the profession of medicine would be fully sufficient for that purpose."

He accordingly, soon after leaving college, placed himself under the care of the late Dr. John Redman, of Philadelphia, a gentleman who had deservedly obtained an extensive share of professional business, and who was justly considered an excellent practitioner. With Dr. Redman, young Rush continued some time, zealously engaged in the acquisition of the several branches of medicine. At that day, however, no institution for the purpose of medical instruction was established in Philadelphia, and his thirst for knowledge being rather excited than gratified, with what he had learned from his preceptor, he formed the resolution of going abroad, in order to avail himself of those advantages which were not within his reach in this country. The University of Edinburgh, at that time, was at the zenith of its reputation, and justly boasted of its able professors, among whom were the elder Munro, the elder Gregory, Dr. Cullen, and Dr. Black. Thither Rush repaired, and was graduated M.D. in 1768, after having performed the usual collegiate duties with much honour, and published his inaugural dissertation *De Concoctione Ciborum in Ventriculo.* In this performance he candidly acknowledged himself indebted, for many of the opinions which he advanced, to his distinguished teacher, Dr. Cullen.

About the period of Dr. Rush's return to his native country, the first attempt was made in Philadelphia for the organization of a medical school. Lectures on anatomy and surgery had indeed been delivered, in that city, in 1763 and 1764, to a small class of pupils, by the late Dr. William Shippen, who, two years previous, had returned from Europe, where he had completed his education under the direction of the celebrated Dr. William Hunter; and, in 1765, Dr. John Morgan, also gave instruction on the institutes of medicine and the practice of physic. Three years after this, the venerable Dr. Kuhn, who had been a pupil of the illustrious Linnæus, and had preceded Dr. Rush in his medical honours at Edinburgh only one year, was made the Professor of Botany and the Materia Medica. To this list of teachers, Dr. Rush himself was added as Professor of Chemistry, immediately upon his arrival from England in 1769. Such was the first organization of the medical college of Philadelphia.

That Dr. Rush had, in an eminent degree, the qualifications of a teacher, and discharged with exemplary fidelity the important duties belonging to the elevated station to which he was chosen, the popularity attending his lectures, the yearly increase in the number of his hearers, and the unexampled growth of the college with which he was connected, bear ample testimony. Shortly after this period, he was elected a fellow, and also one of the curators of the American Philosophical Society.

While Dr. Rush was thus engaged in the active pursuits of his profession, the dispute of the then American colonies with Great Britain arose. Considering the claims of the British government unjust, he entered with warmth into the defence of the rights of his countrymen. His talents were already well known, and the fullest confidence was placed in his integrity and patriotism. The crisis demanded his services; and in the year 1776 he was chosen a member of congress for the state of Pennsylvania, and on the 4th of July, with eight other delegates from that state, he signed the instrument of independence. Upon the 11th day of April, 1777, he was appointed surgeon general of the military hospital in the middle department. His colleague in the medical school, Dr. Shippen, on the same day was appointed director general of all the military hospitals for the armies of the United States, and Dr. J. Jones was made physician general of the hospital in the middle department. The office of surgeon general was not long held by Dr. Rush. He indeed fulfilled the duties of his appointment with all the promptitude suitable to the exigency of the times; but as he had more particularly directed his studies to the practice of physic, and deemed himself better prepared to watch over the concerns of the medical department, he gave a decided preference to a station of that kind. His wishes were soon granted; for upon the 1st of July, 1777, he was created physician-general of the hospital, in the middle department, in the room of Dr. Jones.

On the 6th of February ensuing, Dr. Rush resigned the station of physician-general, and Dr. William Brown was appointed in his place. We shall here forbear any remarks upon Dr. Rush's resignation, nor shall we offer any conjectures as to the causes which led to it. We are certain he did not quit the public service on account of any difficulties incident to the office, or from any want of inclination to promote, by every honourable means, the cause of his country. Serious disputes had arisen among the heads of the medical staff, and there existed many heavy charges of impropriety of conduct on the part of the managers of the hospital stores, which eventually terminated in an investigation by a court of inquiry. The character of Dr. Rush remained unsullied, and being determined to remain free, even from the suspicion of mal-conduct, he totally withdrew himself from the hospital department.

Dr. Rush, however, still continued to take an active part in the politics of the state to which he belonged. The original government of Pennsylvania is known to have been perfectly unique in its form, and the constant source of incalculable mischief. The house of representatives, chosen annually by the people, and on which there was no check, was the sole legislative power: and each succeeding assembly often made it their business to undo all that their predecessors had done. This kind of government was justly reprobated by Dr. Rush, and the necessity and wisdom of a reformation in it was too apparent not to be attempted. Dr. Rush, and many other distinguished abettors of the cause, had, soon after, the satisfaction of seeing a new form of government established in Pennsylvania, by a general convention of the people.

He who has acquired a high reputation in pursuits which do not belong to his profession, is doubly obligated to establish a character as a successful cultivator of that art or science to which his primary attention may have been directed. Of this circumstance Dr. Rush was fully aware: he was already acknowledged an able teacher and practitioner of medicine; but he had also devoted a considerable portion of his time to politics and legislation; and well knowing that the science of medicine afforded ample room for the deepest researches of human intellect, and abundant opportunity for the display of the greatest talents, he formed the resolution of retiring from political life, and of devoting the remainder of his days, with increased ardour, to his profession. He was still further induced to this resolution, from the consideration of the state of medicine in his native country at that time, which, it is scarcely necessary to remark, was in a very low condition. Happy for medical science and the interests of humanity, that he so early formed such a resolution, and that he was so steady, uniform, and indefatigable in the accomplishment of it.

During the long and brilliant career of Dr. Rush's life, from this time to its termination, he may be considered as exclusively occupied in duties pertaining to his profession, and not unlike another Howard, in "surveying the mansions of sorrow and pain," and in mitigating and removing the distresses of all within his power. His biography, therefore, like that of most other scientific men, consists chiefly in a history of his professional labours. How numerous and important his services, as an author, have been, will be readily seen from a brief detail of his writings, which we shall attempt to give, as nearly as practicable, in chronological order.

The first fruits of his professional labours, as an author, was an account of the effects of the Stramonium or thorn apple: this appeared in the year 1770, and was published in the Transactions of the American Philosophical Society, vol. 1st. The same year he addressed a Letter, on the Usefulness of Wort in ill-conditioned Ulcers, to his friend Dr. Huck, of London, which was published in the Medical Observations and Inquiries of London, vol. 4th. In 1774 he read, before the Philosophical Society, his interesting Inquiry into the Natural History of Medicine among the Indians of North America, which formed the subject of an anniversary oration. He this year again addressed another Letter to Dr. Huck, containing some Remarks on Bilious Fevers, which was printed in the London Medical Observations and Inquiries, vol. 5th. To this succeeded his Account of the Influence of the Military and Political Events of the American Revolution upon the Human Body, and Observations upon the Diseases of the Military Hospitals of the United States, which his situation in the army eminently qualified him to make. In 1785, he offered to the Philosophical Society of Philadelphia an Inquiry into the cause of the Increase of Bilious and Intermitting Fevers in Pennsylvania; published in their Transactions, vol. 2d.; and soon after, in quick succession, appeared Observations on Tetanus, an Inquiry into the Influence of Physical Causes upon the Moral Faculty, Remarks on the Effects of Ardent Spirits upon the Body and Mind, and his Inquiry into the Causes and Cure of the Pulmonary Consumption. About this time also, appeared his paper entitled Information to Europeans disposed to migrate to the United States, in a Letter to a friend in Great Britain; a subject which had already occupied the attention of Dr. Franklin, but which Dr. Rush considered still further deserving notice, on account of the important changes which the United States had lately undergone. To this paper followed his Observations on the Population of Pennsylvania, Observations on Tobacco, and his Essay on the Study of the Latin and Greek Languages, which was first published in the American Museum of Philadelphia. This last-mentioned paper, which has been the fertile topic of much animadversion, was, with several other essays of Dr. Rush, and his Eulogiums on Dr. Cullen and the illustrious Rittenhouse, the former delivered in 1790, the latter in 1796, embodied in an octavo volume, entitled, Essays, Literary, Moral, and Philosophical, and published in 1728.

In 1791, the Medical Colleges of Philadelphia, which, on account of certain legislative proceedings, had existed as two distinct establishments since the year 1788, became united under the name of the University of Pennsylvania; and Dr. Rush was appointed to the chair of the professorship of the Institutes of Medicine and Clinical Practice. He now gave to the public his Lectures upon the Cause of Animal Life. The same year he presented the Philosophical Society his Account of the Sugar Maple Tree of the United States, which was published in their Transactions, volume 3d.; and in 1792, Observations, intended to favour a supposition that the Black Colour of the Negro is derived from Leprosy, published in their Transactions, volume 4th.

The year 1793 is memorable in the medical annals of the United States, from the great mortality occasioned by the yellow fever, which prevailed in the city of Philadelphia; and the history of that epidemic, which was published by Dr. Rush in 1794, cannot be too highly valued, both on account of his minute and accurate description of the disease, and the many important facts he has recorded in relation to it. It was comprised in one volume octavo, and has undergone several editions, and been extensively circulated in the Spanish and in the French Language. About this period, also, he offered to the medical world his Observations on the Symptoms and Cure of Dropsy in general, and on Hydrocephalus Internus; an Account of the Influenza, as it appeared in Philadelphia in 1789, 1790, and 1791; and Observations on the State of the Body and Mind in Old Age. In 1797 came out his Observations on the Nature and Cure of Gout, and on Hydrophobia; an Inquiry into the Cause and Cure of the

Cholera Infantum; Observations on Cynanche Trachealis, &c.

It is proper to state, as connected with the literary labours of Dr. Rush, that in 1788, many of his medical papers were collected together, and that he offered them to the public under the title of Medical Inquiries and Observations, volume first. These he from time to time continued, embracing most of the writings above enumerated, besides Observations on the Climate of Pennsylvania, and some others, until a fifth was completed in 1798. In 1801, he added to his character as a writer, by the publication of six Introductory Lectures to a Course of Lectures upon the Institutes and Practice of Medicine, delivered in the University of Pennsylvania. In 1804 a new and corrected edition of his Medical Inquiries, &c. was printed in four volumes octavo. In 1806 he also published a second edition of his Essays. In 1809, such was the demand for the Medical Inquiries and Observations, he again revised and enlarged the work throughout, and enriched the medical profession with a third edition. In this edition he continued his several histories of the yellow fever, as it prevailed in Philadelphia, from 1793 to 1809. It also contained a Defence of Blood-letting, as a Remedy for certain Diseases; a View of the comparative State of Medicine in Philadelphia between the years 1760 and 1766, and the year 1809; an Inquiry into the various sources of the usual forms of Summer and Autumnal Diseases in the United States, and the means of preventing them; and the recantation of his opinion of the contagious nature of the Yellow Fever.

He now formed the idea of selecting some of the best practical works for republication in America; and in order to render them more useful, of adding to them such notes as might the better adapt them to the diseases of his own country. His editions of Sydenham and of Cleghorn were published in 1809, and in 1810 appeared those of Pringle and Hillary. In 1811 appeared a volume of Introductory Lectures, containing those he had formerly published, with ten others, delivered at different years before his class, and also two upon the Pleasures of the Senses and of the Mind. His work upon the Diseases of the Mind, which had long and ardently been looked for, was next added to his writings. It appeared towards the close of 1812, in one volume octavo. The last effort of his pen was a Letter on Hydrophobia, containing additional reasons in support of the theory he had formerly advanced, as to the seat of the disease being chiefly in the blood-vessels. It was addressed to Dr. Hosack, and written not many days before his fatal illness.

While thus assiduously engaged in enriching medical science with the valuable fruits of his long and extensive experience, and in the active discharge of the practical duties of his profession, he was, on the evening of the 13th of April, seized with symptoms of general febrile irritation, which were soon accompanied with considerable pain in his chest. His constitution was naturally delicate; he had acquired, from previous illness, a predisposition to an affection of his lungs. He lost a moderate quantity of blood, by which he felt himself considerably relieved. But his strength was not sufficient to overcome the severity of his complaint: the beneficial effects resulting from the most skilful treatment were but of a temporary duration. His disease rapidly assumed a typhus character, attended with great stupor and a disinclination to conversation. In other respects, however, he retained his faculties, and the perfect consciousness of his approaching dissolution. On Monday evening ensuing, after a short illness of five days, and in the 69th year of his age, he ended his truly valuable and exemplary life. His death was the subject of universal lamentation, and he was followed to the grave by thousands, who assembled to bear testimony of his excellence.

In January, 1776, he married Miss Julia Stockton, daughter of the Hon. Judge Stockton, of New Jersey, a lady of an excellent understanding, and whose amiable disposition and cultivated mind eminently qualified her as the companion of Dr. Rush. Thirteen children were the fruits of their marriage, nine of whom still survive. Two of these are chosen to offices of high respectability in the general government of the United States.

It were no easy task to do adequate justice to the great talents, the useful labours, and the exemplary character of Dr. Rush. From the preceding sketch, it is presumed, some idea may be formed of his incessant devotedness to the improvement of that profession of which he was so bright an ornament. His merits, as a practitioner, are too well known to need particular notice: he was fully aware of the great responsibility attached to the medical character, and uniformly evinced the deepest solicitude for the recovery of his patient. His kindness and liberality in imparting aid to those from whom no remuneration was ever to be expected was unbounded, and arose from the generous impulse of his nature, the cordial concern he felt in whatever affected the interests of his fellow creatures. His mind was of a superior order: to a perception naturally ready and acute, he united a discriminating judgment, a retentive memory, which was greatly improved by habits of close attention, a brilliant imagination, and a highly cultivated taste. He possessed a comprehensive understanding; his knowledge was varied and profound, and he eminently excelled in the several departments of his profession. In his assiduity and perseverance in the acquisition of knowledge he had no superior, and few equals. Accustomed to constant and regular exercise, his intellectual powers acquired additional vigour from employment. Notwithstanding the great fatigue he had to undergo in the discharge of the practical duties of a laborious profession, and the constant interruptions to which he was exposed, when engaged in his pursuits as an author, he never for a moment abated of his ardour in the cause of science. His habits of punctuality to every kind of business in which he was employed, added to a judicious arrangement of time for his multifarious occupations, secured to him sufficient leisure for the publication of those works which have given such celebrity to his name.

His writings claim our attention, both on account of their extent and their variety. Instead of being a mere collator of the opinions of others, he was constantly making discoveries and improvements of his own; and from the results of his individual experience and observation, added more facts to the science of medicine than all who had preceded him in his native country. His description of diseases, for minuteness and accuracy of detail, cannot be exceeded, and may safely be regarded as models of their kind. In the treatment of gout, dropsy, consumption of the lungs, and the diseases of old age, he enlarged our views of the animal œconomy, and threw more light upon the peculiar character of these afflicting disorders, than is to be derived from the investigations of any other writer. His volume on the diseases of the mind, in as far as it shows the infinitely varied forms which those ideas exhibit, is a storehouse of instruction. Had his labours been limited to these subjects alone, his character would deservedly have been cherished by future ages. His reputation, however, will permanently depend upon his several histories of the epidemics of the United States, which have rendered his name familiar wherever medical science is cultivated, and will hereafter cause to be inscribed upon the same imperishable column that bears testimony to the merits of Sydenham and Boerhaave, the illustrious name of Benjamin Rush. The respect and consideration which his publications procured for him among his contemporaries, was such, that the highest honours were accumulated upon him in different parts of Europe, as well as in his own country, and he was admitted a member of many of the most distinguished literary and philosophical associations.

There are other qualities which still more entitled Dr. Rush to our respect and esteem. In private life, his disposition and deportment were in the highest degree exemplary. Admired and courted for his intellectual endowments, he riveted to him the affections of all who enjoyed the pleasure of an intimate acquaintance. The affability of his manners, the amiableness of his temper, and the benevolence of his character, were ever conspicuous. He was ardent in his friendships and forgiving in his resentments; and yet, entertaining a due regard for himself and a high sense of honour, he possessed a manly independence of spirit, which disdained every thing mean and servile. He had an extraordinary command of language, and always imparted his thoughts in a peculiarly impressive and eloquent manner. Those who had the happiness to experience the delights of his conversation, will long recollect, with pleasure, his unassuming modesty, and the rich stores of knowledge he poured forth on the most instructive topics. Even when his opinions were solicited, they were given, not as the dictates or admonitions of a superior, but as the kind advice of a friend and equal. He never evinced any of that haughtiness and affectation of importance, which sometimes attaches to men of eminence, and which so materially lessens the pleasures and comforts of social life.

He was a believer in Christianity from an examination of its principles and the deepest conviction. The purity of its doctrines and the excellence of its precepts was a frequent topic of his conversation: its practical influence upon his conduct through life he often acknowledged, and cherished with a fervent hope the animating prospects which it affords. His writings, in numerous places, bear testimony to his Christian virtues; and in a manuscript letter, written a short time previous to his fatal illness, and now before the writer of this imperfect sketch, he candidly declares, that he had "acquired and received nothing from the world which he so highly prized as the religious principles he received from his parents." It is peculiarly gratifying to observe a man so distinguished in a profession in which, by the illiberal, religious scepticism is supposed to abound, directing his talents to the maintenance of genuine piety, and the enforcing of Christian virtue. To inculcate those principles which flow from the source of all truth and purity, and to impart them as a legacy to his children, was an object dear to his heart, and which he never failed to promote by constant exhortation, and the powerful influence of his own example.

---

*To the Editors of the Medico-Chirurgical Journal & Review.*

GENTLEMEN,

THE recent proposal of employing the vapour of boiling tar in the cure of pulmonary consumption, naturally leads to the humiliating reflection, that the practical result of the numerous remedies recommended in that disease, has hitherto served only to expose their futility; and, that having added to the proofs of the fallacy of our conjectural art, they have, in succession, sunk into merited neglect. In adverting to those discouraging events, it is not, however, my intention to express anticipation of a similar fate in respect to the present remedy, or to offer any estimate of its probable degree of efficiency. The question is strictly one of fact; and its decision must be referred to the only competent tribunals—those of time and experience.

In contemplating the origin and progress of a discovery, the mind reverts with interest to any analogous occurrence which may possibly have contributed to elicit the germ of the inquiry, or to promote its advancement to the full bloom of maturity. Without entertaining the most distant wish to derogate from the novelty of the treatment under consideration, I may be permitted to observe, that a practice, very similar in process, was adopted in France (literally by *inspiration,*) more than forty-five years since. What degree of credit it obtained in that country, I know not; but the circumstance is recorded in a very respectable publication. It will be observed, that there is a difference in the choice of the material from which the salutary fume is obtained; sufficient indeed, to afford ample protection to the originality of the present discovery, should further experience prove the claim to be deserving attention.

In the correspondence of Baron de Grimm and M. Diderot with the Prince of Saxe Gotha, in 1771, the following statement occurs.

"An officer, in garrison at Rochefort, wearied with having pursued for a long time, without effect, the usual remedies for an obstinate cold, abandoned them at last, and resumed his ordinary course of life. He soon began to spit blood, and his lungs appeared to be seriously affected; still he persisted in abstaining from his remedies. One day, having bottled off a cask of wine in his cellar, he had half a pound of yellow wax, and half a pound of rosin brought into his room, which he set about heating over the brazier, to seal down the corks of his bottles. This operation having lasted an hour and a half, he thought that he spit more freely, and that his cough was less dry and frequent. It then occurred to him, that this might be the effect of the fumigation he had undergone, and he determined to renew the experiment. He accordingly walked about his room, keeping the doors and windows close shut, in a perfect cloud formed by the smoke, and in four or five days found himself perfectly cured.

He imparted the discovery to the surgeon of the regiment, who, without having any great faith in its efficacy, thought there would be no harm in trying the experiment upon a soldier in the hospital, who was dying of a pulmonary complaint. He had him brought to his house, and made him, at intervals of four hours, undergo a fumigation proportioned to his strength; for being in a very weak state, he might have been suffocated by too strong a smoke. From the second day the patient's cough began to abate, and in six weeks his health was perfectly re-established."

I am, &c.

NAUTA.

*April* 4, 1818.

---

A very curious lamp has recently been constructed upon the principle of Sir Humphrey Davy's late discoveries, which, while it forms a very amusing philosophical experiment, promises to be of considerable use. A cylindrical coil of platina wire, about 1-100th of an inch thick, and containing about ten turns, is placed, part of it round the cotton wick of a spirit lamp, and part of it above the wick; the lamp is then lighted, so as to heat the wire to redness. When the flame is blown out, the vapour of the alcohol will keep the upper part of the wire red hot, as long as there is a supply of alcohol, of which the expenditure is very trifling. The heat of the wire is sufficient to kindle German fungus, or paper prepared with nitre; so that a sulphur match may be lighted when it is required. A wick, composed of twelve threads of the ordinary sized lamp cotton yarn, will require about half an ounce of alcohol to keep it lighted for eight hours. When the wire becomes oxidized, it is necessary to uncoil it, and rub it bright again with fine glass paper.

---

### NEW BOOKS.

Facts and Observations on Liver Complaints and those various and extensive Derangements of the Constitution arising from hepatic irregularity and obstruction; with practical Remarks on the Biliary and Gastric Secretions, and upon other important points essential to health; pointing out a successful mode of Treatment, and illustrated by numerous Cases. The third Edition, with additional practical observations; by John Faithhorn, formerly Surgeon in the Hon. East India Company's Service.

In the press, and speedily will be published, an Essay on the Symptoms, Causes, and Treatment of Inversio Uteri, with a History of the successful Extirpation of that Organ during the chronic Stage of the Disease; by William Newnham, Farnham.

A new edition, considerably improved, of Dr. Withering's Systematic Arrangement of British Plants, with an easy Introduction to the Study of Botany, in 4 vols. 8vo. illustrated by copper plates, is in the press, and will be shortly published.

Modern Maladies and Present State of Medicine. The Anniversary Oration delivered before the Medical Society of London, and published at their request.

---

☞ *On the 1st of July the Medico-Chirurgical Journal will commence a slower march, and assume a larger form. It will be published, from that time, Quarterly, price 3s. 6d. under the sole direction of Dr. James Johnson, and will be substituted for the Medical Register, projected by that Gentleman. A Prospectus, relative to the new arrangement, together with the contents of the first quarterly number, will appear in our next publication.*

---

